# Identifying new targets in the fight against opioids

**DOI:** 10.7554/eLife.109920

**Published:** 2026-01-05

**Authors:** Fernanda Laezza

**Affiliations:** 1 Department of Pharmacology and Toxicology and the Sealy Center for Environmental Health and Medicine, University of Texas Medical Branch Galveston United States

**Keywords:** opioid use disorder, epistatic interactions, regulation of neuronal excitability, Oprm1, Fgf12, Mouse, Rat

## Abstract

Experiments reveal that a time-dependent epistatic interaction influences how mice respond to opioids, and that intracellular fibroblast growth factors also influence opioid sensitivity.

**Related research article** Lemen PM, Zuo Y, Hatoum AS, Dickson PE, Mittleman G, Agrawal A, Reiner BC, Berrettini W, Ashbrook DG, Gunturkun MH, Wang X, Mulligan MK, Browne CJ, Nestler EJ, Telese F, Williams RW, Chen H. 2025. Acute opioid responses are modulated by dynamic interactions of *Oprm1* and *Fgf12*. *eLife* **14**:108845. doi: 10.7554/eLife.108845.

Opioid use disorder is a global public health crisis that continues to have devastating consequences for individuals and society (Mathis et al., 2025). Despite its prevalence, the mechanisms underlying this disorder remain poorly understood, perpetuating stigma and limiting effective treatments. At its core, opioid use disorder is a chronic brain disorder marked by dysregulation of reward circuits, which traps individuals in a cycle of compulsive drug seeking and impaired ability to abstain. Current therapies are limited to preventing overdoses, and there is a lack of therapeutics that can modify the mechanisms underlying the disorder.

Now, in eLife, Hao Chen, Robert Williams and co-workers – including Paige Lemen of the University of Tennessee Health Science Center (UTHSC) as first author – report a major advance in our understanding of the molecular architecture of opioid use disorder (Lemen et al., 2025). Using a systems genetics approach in mouse models, Lemen et al. generated high-resolution time-series data for morphine-induced behaviors, and then mapped these traits to specific genetic loci.

Two key modulators emerged: a gene on chromosome 10 called *Oprm1*, which codes for a well-known opioid receptor; and a gene on chromosome 16 that codes for a signaling protein called fibroblast growth factor 12 (Fgf12), which has not previously been linked to opioid use disorder. Complementary studies revealed that both genes are highly expressed in neurons that express a dopamine receptor called DRD1: these neurons are critical components of the reward circuit in the central nervous system.

Lemen et al. – who are based at UTHSC and other institutions across the US – also applied a computational network analysis, which supported a model involving enzymes called MAP kinases, and a voltage-gated sodium channel called Nav1.2 (Figure 1). These enzymes regulate neuronal activity and interact with Fgf12 (Schoorlemmer and Goldfarb, 2001), while the Nav1.2 sodium channel controls neuronal excitability and is a known binding partner of Fgf12 (Wildburger et al., 2015). Moreover, mutations in Fgf12 lead to dysfunction of Nav1.2, and have been linked to epilepsy and a number of encephalopathies (Siekierska et al., 2016; Trivisano et al., 2020; Pierret et al., 2025; Seiffert et al., 2022). This model is intriguing as it reveals the central role of *Oprm1*, and the importance of interactions between proteins that are involved in the regulation of neuronal excitability (that is, between MAP kinases and the Nav1.2 sodium channel).

**Figure 1.**
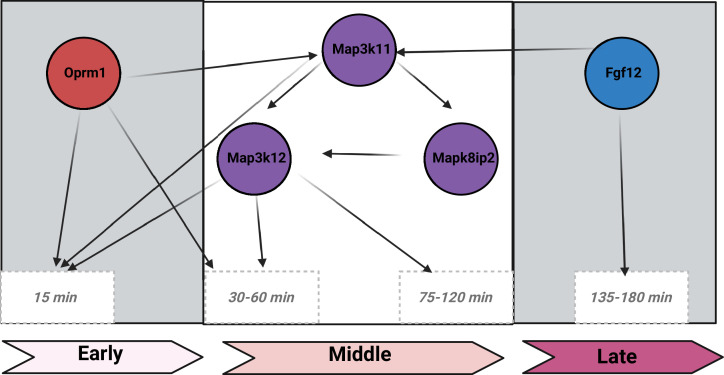
The Oprm1–Fgf12 network in opioid use disorder. Lemen et al. identified two genes that are associated with opioid use disorder: *Oprm1*, which is located chromosome 10 and codes for a well-known opioid receptor; and *Fgf12* on chromosome 16, which codes for a signaling protein with no previously known links to opioids. The influence of morphine on locomotor responses (that is, movement) varies with time (horizontal axis). From about 15 minutes after the mice were injected with opioid until ~30–60 minutes after injection (early phase), Oprm1 is the main influencer of movement. The two black arrows pointing from Oprm1 represent its direct influence on locomotor response, while the arrow pointing toward Map3k11 at about 60 minutes after injection, indicates that Oprm1 initiates signaling through the MAPK pathway. During this period (middle phase), Oprm1 functions as a causal driver by activating Map3k11, which in turn modulates Mapk8ip2 and Map3k12, ultimately influencing movement. Oprm1 and Fgf12 subsequently interact with the kinase Map3k11, which interacts with Map3k12 (either directly or via Mapk8ip2), to influence the locomotor response. Then, between 135 and 180 minutes after injection (late phase), Fgf12 exerts a direct effect on locomotor response, independent of Oprm1. It becomes the dominant regulator of movement, marking a temporal shift from early cooperative interaction through epistasis to late Fgf12-specific control. Lemen et al. developed this causal hypothesis using the Bayesian network framework that is available in GeneNetwork.org.

Another compelling finding was that there was a strong epistatic interaction between *Oprm1* and *Fgf12* during a short time window that started 45 minutes after morphine had been administered, and ended 30 minutes later. This dynamic interplay suggests opioid sensitivity is governed not by static genetic effects, but by evolving molecular networks that are orchestrated by these genes. The translational relevance of this is further underscored by evidence that the Oprm1–Fgf12 network is enriched in human GWAS data for substance use disorders.

By identifying *Fgf12* as a novel candidate gene and highlighting its epistatic interaction with *Oprm1*, this study sets the stage for a paradigm shift in our approach to opioid use disorder, from focusing solely on receptor-level pharmacology to embracing complex genetic and signaling networks. It also reinforces the emerging role of intracellular fibroblast growth factors in neuronal excitability (Libé-Philippot et al., 2023), in neuropsychiatric conditions (Dvorak et al., 2025) and substance use disorders (Dvorak et al., 2023), highlighting converging mechanisms where genes that fine-tune neuronal firing act as key regulators of reward-related behaviors and addiction.

Future studies should explore different aspects of these genetic interactions. Do they translate to the regulation of protein-protein interaction complexes in cells? Do they influence long-term adaptations to repeated opioid exposure? And do they extend to other substance use disorders? Integrating behavioral phenotyping with computational modeling – as Lemen et al. did here – will be critical to efforts to unravel the temporal dynamics of these networks, and to drive mechanistic hypotheses that can guide new therapeutic strategies for opioid use disorder.

Ultimately, translating these discoveries into clinical practice will require collaboration across genetics, neuroscience and pharmacology. By illuminating new molecular targets and the interactions between them, this study is creating a roadmap for therapies that address the root mechanisms of opioid use disorder, rather than its symptoms.
